# Observation on the efficacy of skin closure by skin staplers in extended L-shaped incisions for calcaneal fractures

**DOI:** 10.1186/s13018-026-06690-x

**Published:** 2026-02-03

**Authors:** Zhen Ma, Jingxin Chen, Xuan Nan, Yuan Li, Dong Li, Fushan Hou

**Affiliations:** 1 Shanxi Medical University, The Second Hospital of Shanxi Medical University, Taiyuan, China; 2 Department of Orthopedics, Second Hospital of Shanxi Medical University, 030000 Taiyuan, China

**Keywords:** Calcaneal fracture, Skin stapler suture, Allgower-Donati suture, Foot and ankle

## Abstract

**Objective:**

This study aims to evaluate the effectiveness of skin staplers for wound closure following extended L-shaped incisions in the treatment of calcaneal fractures.

**Methods:**

A randomized controlled trial was performed on 82 calcaneal fracture cases (Sanders types III-IV) that underwent extended L-shaped incisions at the Second Hospital of Shanxi Medical University from June 2022 to March 2025. Out of these, 60 cases met the inclusion and exclusion criteria. A non-blinded, open, randomized controlled trial was conducted, assigning patients to either the stapler group (*n* = 28) or the Allgower-Donati group (*n* = 32). The key parameters assessed included wound closure time, the area of postoperative inflammatory reaction, changes in skin temperature at the incision corners, and wound healing grades. These parameters were then compared between the two groups.

**Results:**

At the 2-week postoperative follow-up, when sutures were removed, no significant differences were found in terms of inflammatory reaction area, skin temperature changes, or wound healing grades between the two groups. However, the stapler group demonstrated a notably shorter wound closure time (10.89 ± 2.87 min) compared to the Allgower-Donati group (20.44 ± 2.01 min).

**Conclusion:**

The use of skin staplers for wound closure resulted in healing outcomes that showed no statistically significant differences from those achieved with the Allgower-Donati suturing technique in extended L-shaped incisions for calcaneal fractures. Importantly, the skin stapler method significantly reduces wound closure time (*P* < 0.05), which can lead to shorter overall surgical durations and a decreased risk of wound infections.

## Introduction

Calcaneal fractures represent approximately 1–2% of all fractures and account for over 60% of foot fractures, making them the most prevalent type of tarsal fractures [1]. Postoperative wound complications are a significant concern in the treatment of these fractures [2–5]. Although techniques like the sinus tarsi incision and minimally invasive closed reduction have decreased the incidence of complications, the L-shaped incision continues to be the primary surgical approach for complex calcaneal fractures (Sanders types III-IV) due to its effectiveness in achieving proper reduction. However, this method presents significant postoperative challenges, such as poor wound healing and skin necrosis. These complications arise from the limited soft tissue coverage and insufficient blood supply in the posterior and lateral regions of the calcaneus [6]. Consequently, the wound experiences persistent high tension, which hinders healing and leads to microcirculatory impairment by compressing local blood vessels [7]. This impairment restricts the delivery of essential nutrients and immune cells, thereby heightening the risk of skin complications [8–10]. Ultimately, inadequate wound healing can result in infection and skin necrosis.

The Allgöwer⁃Donati suturing technique, a modified vertical mattress method introduced by Allgöwer and Donati in the 1960s, is particularly effective for closing skin incisions. This technique employs an intradermal suturing path that passes through the dermis without impacting the epidermis, which minimizes suture exposure and reduces the risk of postoperative infection and hypertrophic scarring. As a result, the Allgöwer-Donati suturing produces favorable cosmetic outcomes, making it especially suitable for incisions on the face, hands, and feet [11]. This method also optimizes microcirculatory perfusion in the incision area, effectively promoting tissue repair [12]and significantly enhancing healing outcomes for extended L-shaped incisions in calcaneal fractures. Consequently, the Allgöwer-Donati suturing technique has gained widespread acceptance for the closure of such incisions in the treatment of calcaneal fractures [12, 13].

Wound healing outcomes are affected not only by suturing techniques but also significantly by the duration of the surgical procedure. Prolonged operation times result in increased tissue exposure, which can exacerbate tissue damage and tension [14]. Consequently, there is a pressing need for instruments that can facilitate rapid closure of incisions. Recent developments in medical technology have introduced the medical skin stapler, a novel tool designed for quick and efficient skin closure. The benefits of using medical skin staplers include expedited wound closure, shortened operative time [15], minimal foreign body reactions to metal staples [16], and enhanced wound healing with reduced cicatricial hyperplasia. Although skin staplers offer the clear advantage of reducing wound closure time, concerns are often raised regarding the safety of wound healing in the calcaneal region due to its unique anatomical characteristics and limited soft-tissue coverage.

This study aims to compare and assess wound healing outcomes between the Allgöwer⁃Donati suturing technique and skin closure using a skin stapler for extended L-shaped incisions, with the goal of providing valuable insights for clinical practice.

## Data and method

### Inclusion and exclusion criteria

*Inclusion criteria*: (1) CT-confirmed Sanders Type III or IV calcaneal fractures; (2) Age range: 18–65 years; (3) Interval between injury and surgery not exceeding 2 weeks.

**Fig. 1:**
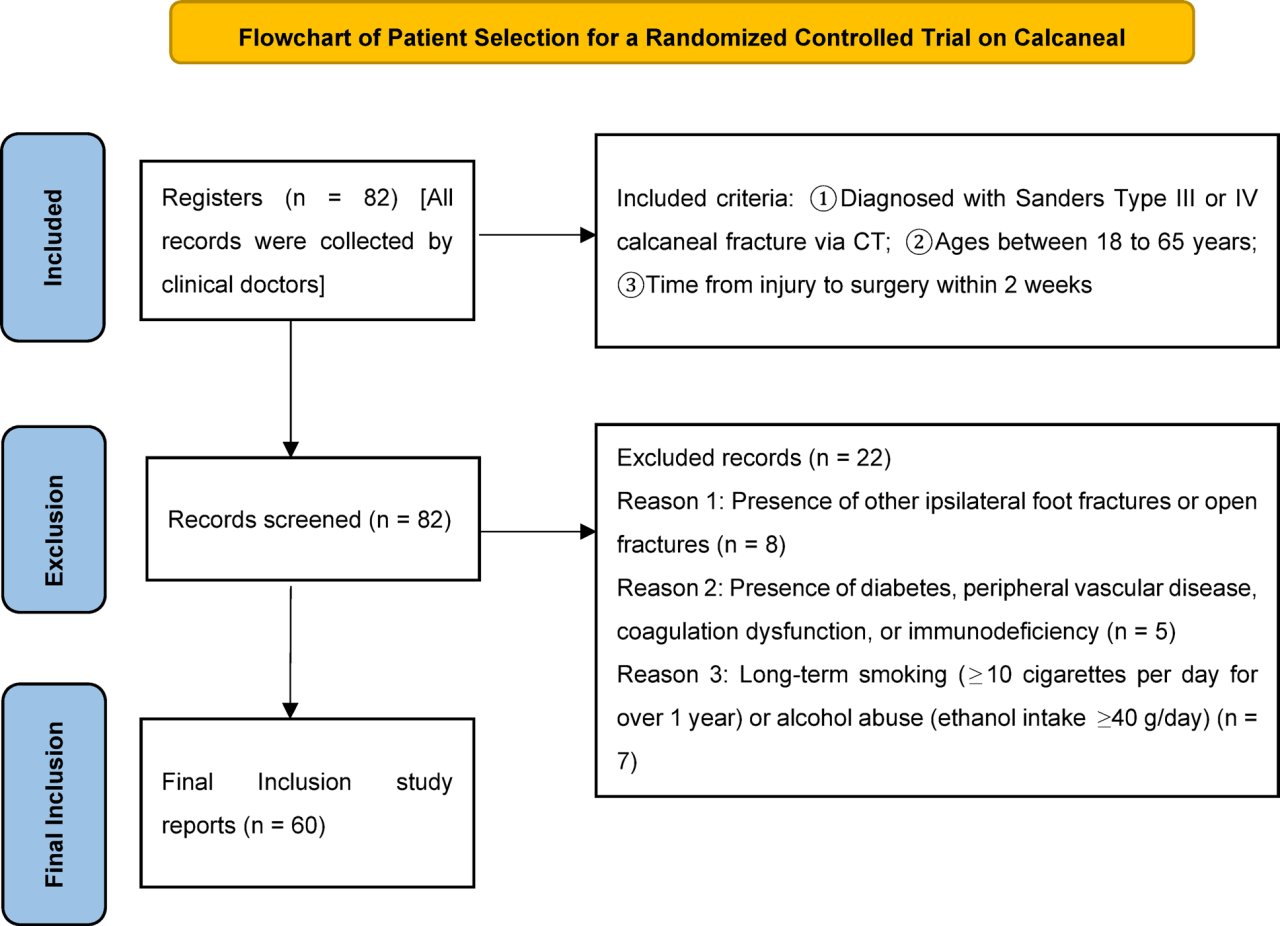
Patient enrollment flowchart. This diagram illustrates the process of screening, exclusion, and final inclusion of participants in the randomized controlled trial

*Exclusion criteria*: (1) Presence of other fractures or open fractures in the same foot; (2) Diagnosed diabetes, peripheral vascular diseases, coagulopathy, or immunodeficiency (e.g., long-term use of immunosuppressants); (3) Chronic smoking (≥ 10 cigarettes daily for over 1 year) or alcohol abuse (ethanol intake ≥ 40 g/d); (4) Coexisting severe hepatic or renal impairment, malignant tumors, or connective tissue diseases; ⑤ Females who are pregnant or breastfeeding. As shown in Fig. 1.

### General information

This study was conducted as a randomized controlled trial. Due to the inherent differences in the visual characteristics of the two closure techniques, blinding of both the surgeons and the patients was not feasible. To minimize potential assessment bias, postoperative outcome evaluators were blinded to group assignments whenever possible. Furthermore, the data analysts maintained blinding throughout the statistical analysis process.

This randomized controlled trial enrolled 82 patients with Sanders type III–IV calcaneal fractures between June 2022 and March 2025. Of these, 60 eligible participants were randomized to either the Stapler closure group (*n* = 28) or the Allgöwer-Donati closure group (*n* = 32). Due to the inherent visual differences between the two closure techniques, blinding of the surgeons and patients was not feasible. To minimize assessment bias, postoperative outcome assessors and data analysts were blinded to group allocation throughout the evaluation and statistical analysis. Detailed randomization procedures are available from the authors upon request. The study received approval from the relevant institutional ethics committee and was registered accordingly.

In the Stapler group, there were 16 males (57.1%) and 12 females (42.9%), with ages ranging from 18 to 65 years and a mean age of 52.8 years (± 9.42). The group included 13 patients classified as IIIAB, 10 as IIIAC, and 5 as IV. In the Allgöwer-Donati group, there were 18 males (56.3%) and 14 females (43.7%), also aged 18–65 years, with an average age of 51.00 years (± 7.98). This group comprised 10 patients classified as IIIAB, 18 as IIIAC, and 4 as IV. No statistically significant differences were found between the two groups regarding gender, age, or fracture classification (*P* > 0.05 in all instances, see Table 1).

**Table 1:** General information of the stapler group and the Allgöwer-Donati group (Comparison of demographic and fracture characteristics between the two groups)

	Number of cases	gender		Age (years) $\:\stackrel{-}{\boldsymbol{x}}$±s	fracture classification		
		M	F		IIIAB	IIIAC	IV
The Stapler group	28	16	12	52.8 ± 9.42	13	10	5
The Allgöwer⁃Donati group	32	18	14	51.00 ± 7.98	10	18	4
statistical quantity	/	Χ^2^ = 0.005		t = 0.846	Χ^2^ = 2.53		
P value	/	0.9444		0.401	0.282		

All patients suffered calcaneal fractures due to falls from a height. Clinical signs included swelling, ecchymosis, bruising, and tenderness localized to the heel area, with some cases presenting palpable bone crepitus. Each patient underwent preoperative imaging, including X-rays (lateral and axial views) and CT scans for three-dimensional reconstructions. The imaging results revealed calcaneal varus deformity, longitudinal splitting, and compression, with a central compression fracture fragment identified. Two radiologists standardized the classification following analysis. This study received approval from the Ethics Committee of our hospital, and all patients provided informed consent before participation.

### Surgical method

#### Anesthesia and positioning

All patients received combined spinal and epidural anesthesia. Those with unilateral calcaneal fractures were placed in a lateral decubitus position, while patients with bilateral fractures underwent staged surgical procedures. A consistent team of experienced surgeons performed all operations. An inflatable tourniquet was applied to the groin of the affected limb, and an extended L-shaped incision was made on the lateral aspect of the calcaneus.

#### Open reduction and internal fixation of calcaneal fractures

**Fig. 2:**
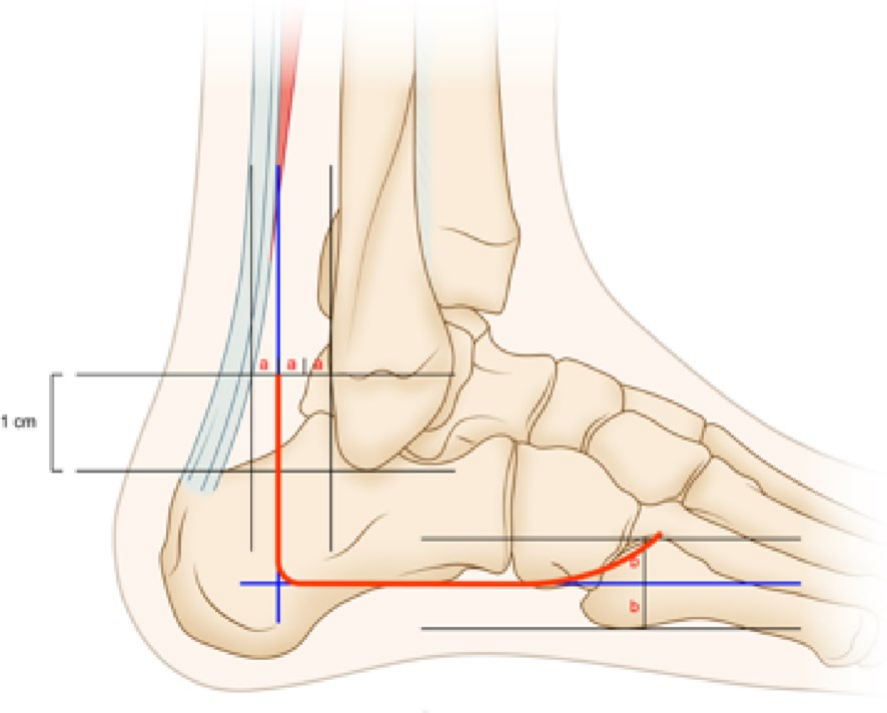
Diagram of the lateral expansion “L” incision of the calcaneus

**Fig. 3:**
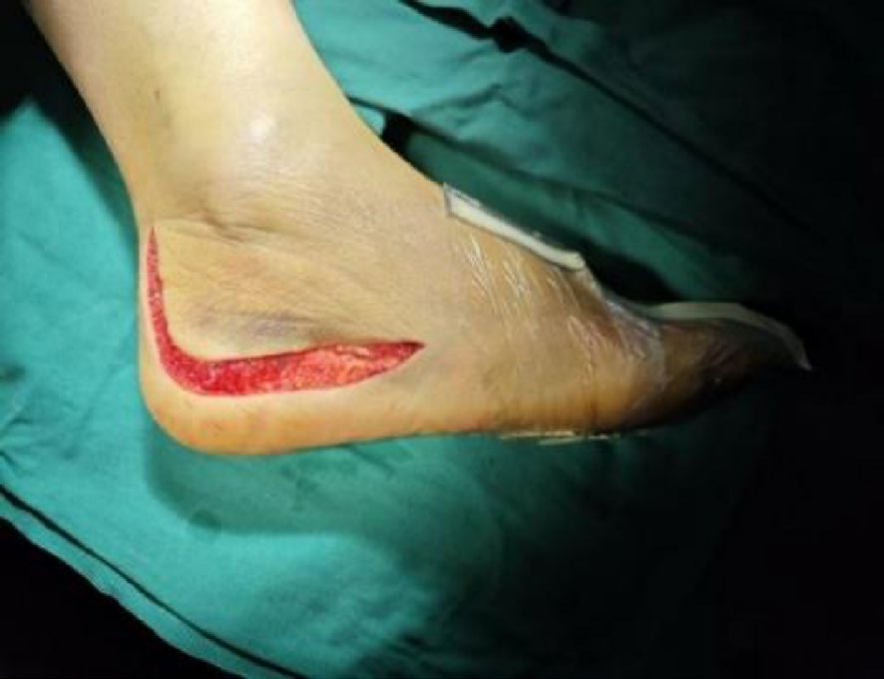
Wound photo of the lateral expansion “L” incision on the calcaneus

The proximal incision begins at the mid-posterior one-third of the line connecting the posterior border of the fibula to the anterior border of the Achilles tendon, positioned 1 cm above the tip of the lateral malleolus. It extends downward to the dermal junction, then transitions horizontally toward the base of the fifth metatarsal, curving slightly upward as illustrated in Figs. 2 and 3. A full-thickness incision is made through both the skin and deep fascia. Using a sharp scalpel, create a full-thickness skin flap along the bone for optimal protection. It is crucial to carefully preserve the peroneal tendon and peroneal nerves during this procedure, as their integrity is essential for adequate exposure of the subtalar and calcaneal joints. The entire flap should then be flipped superiorly and secured with three 2.0 K-wires attached to the cuboid bone, talar neck, and talar body, serving as a dynamic protective barrier. This positioning ensures complete exposure of the lateral calcaneal wall and subtalar articular surface. Under direct visualization, elevate the lateral wall using a periosteal elevator to lift and elevate the articular surface, restore the calcaneal width, Gissane angle, and Böhler angle, and correct any varus deformity. Apply temporary fixation with K-wires. Once fluoroscopic imaging confirms satisfactory positioning, proceed to place the plate, drill holes, measure depth, and insert screws.

#### Incision closure

Intraoperative fluoroscopy confirmed satisfactory fracture reduction, along with appropriate positioning and length of the plate and screws. The wound was thoroughly irrigated, and a No. 10 drainage tube was inserted postoperatively. The patients were subsequently randomized to receive one of the two skin closure techniques, as described below. In both groups, the deep fascia was consistently closed with interrupted sutures using 3 − 0 absorbable Vicryl^®^ suture (Ethicon Inc., a Johnson & Johnson company, Somerville, NJ, USA).

**Fig. 4:**
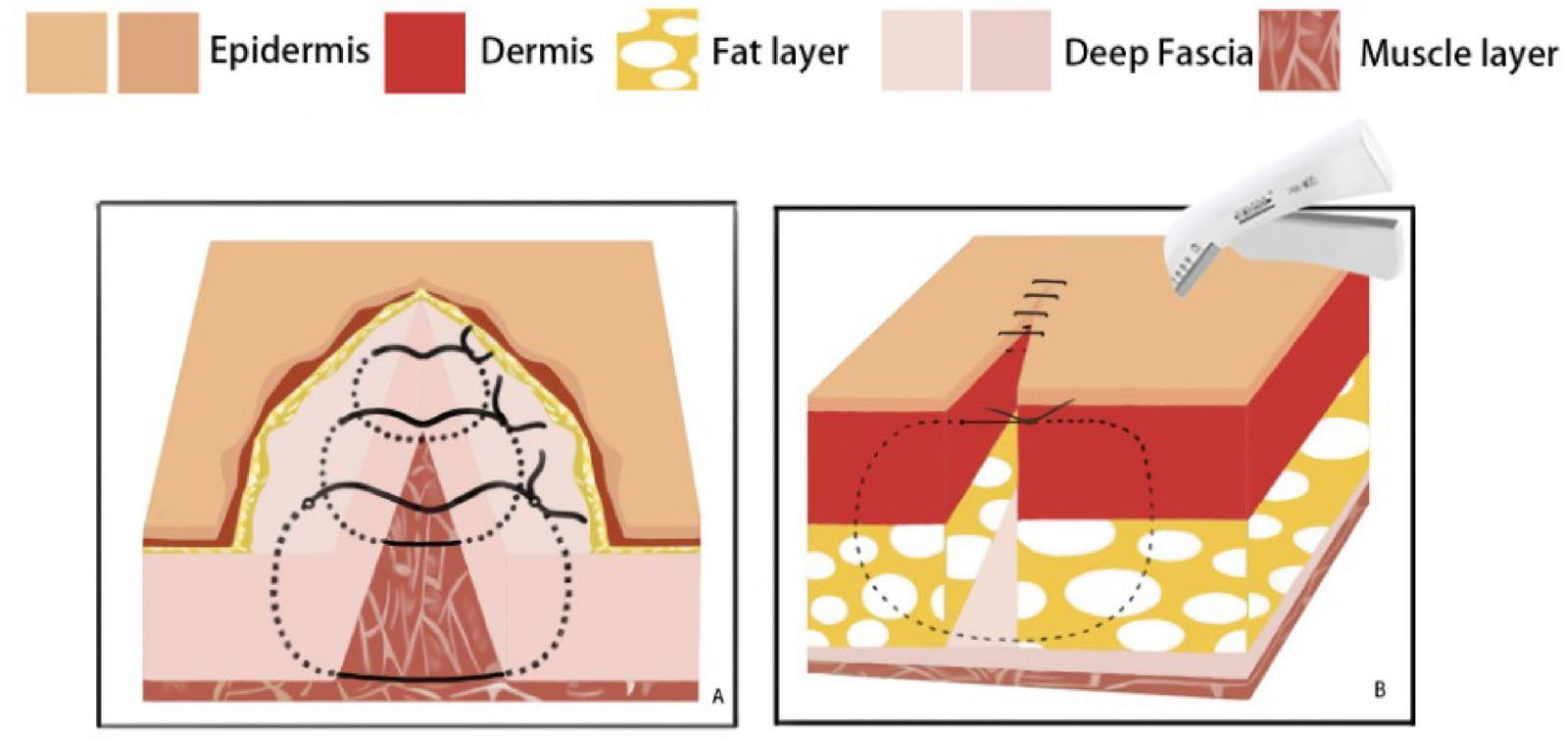
The skin grafting unit first uses 3-0 Vicryl® for the full-thickness deep fascia suture, and then directly performs skin grafting

**Fig. 5:**
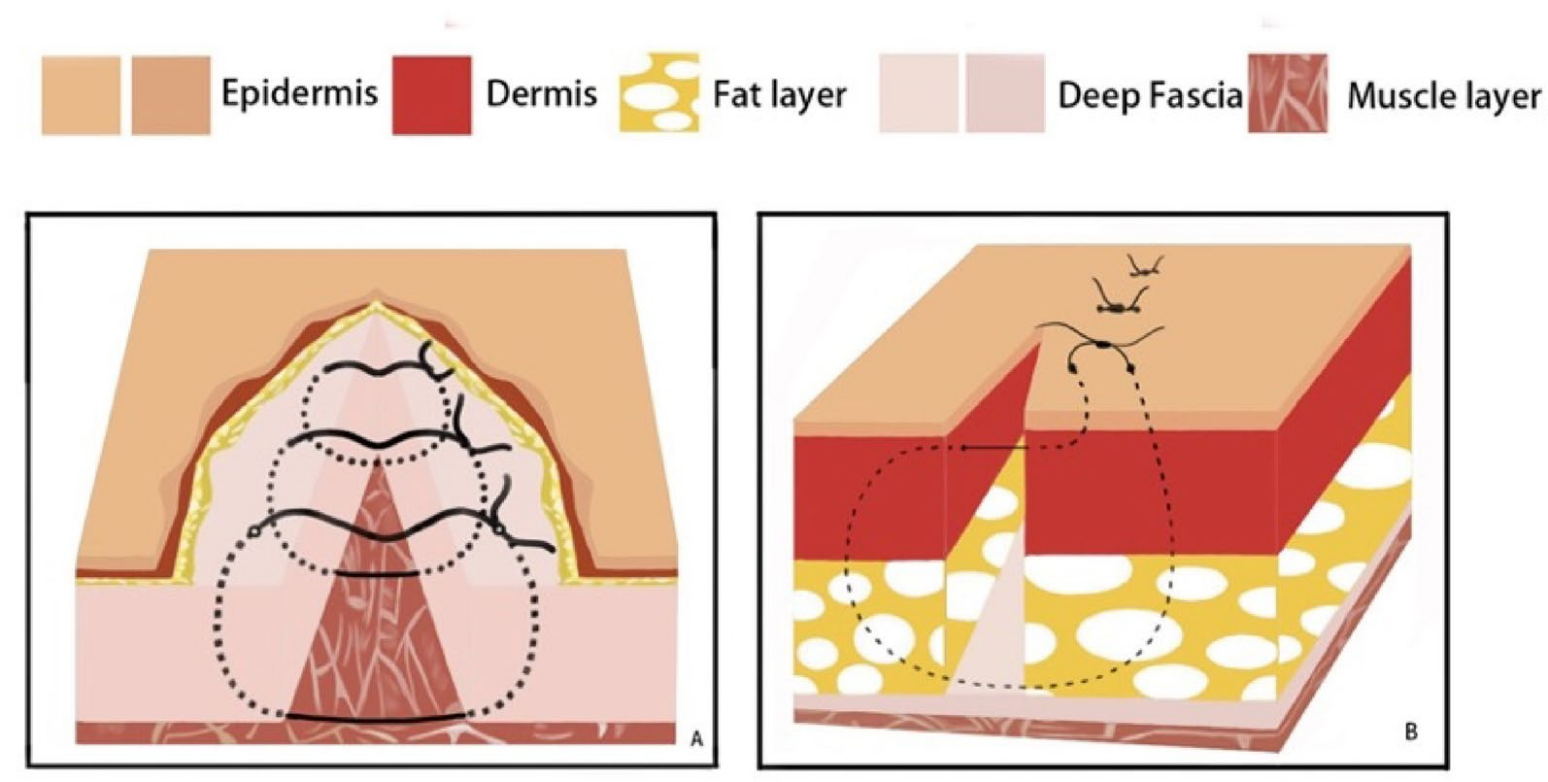
Schematic diagram of Allgöwer-Donati suture method: **A** Layer 1: First, perform 3-0 Vicryl® intermittent suture on the deep fascia, with an interval of approximately 8-10 mm. **B** Layer 2: Use 4-0 Polynil line. Allgöwer-Donati suture method

1. *Stapler Group Closure*: After fascial closure, the skin incision was directly apposed and closed using a skin stapler, as demonstrated in Fig. 4.
2. *Allgöwer-Donati Group Closure*: After fascial closure, the skin was closed using the Allgöwer-Donati suturing technique with 4-0 Prolene sutures. These sutures were inserted plantarly through the skin, traversed subcutaneously to the opposite side, and exited at the dermal layer along the contralateral skin margin without penetrating the epidermis. The suture intervals were maintained at 8–10 mm, as illustrated in Fig. 5.

**Fig. 6:**
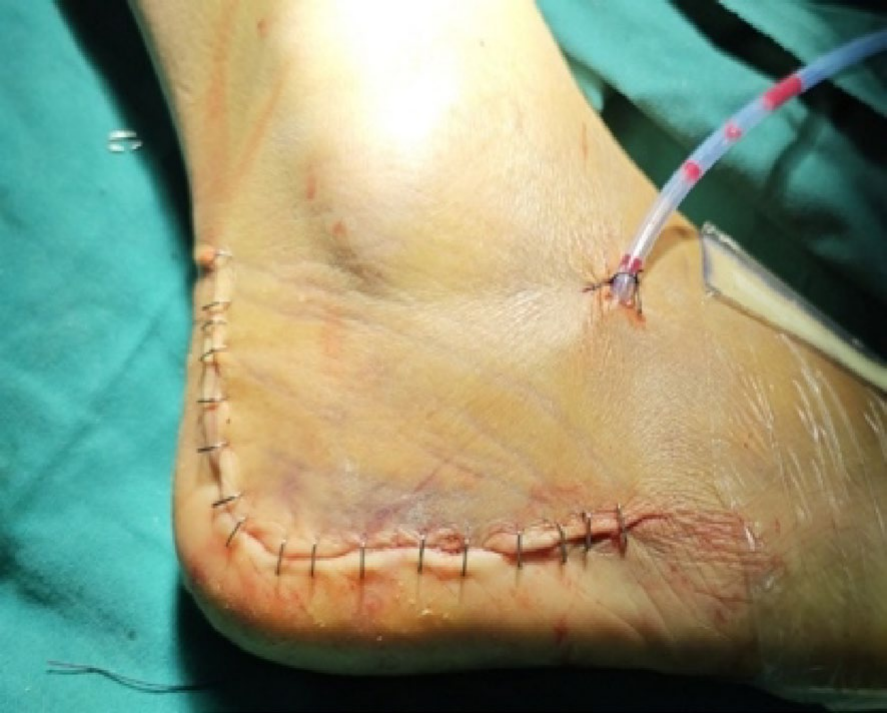
Stapler closure for incision closure

**Fig. 7:**
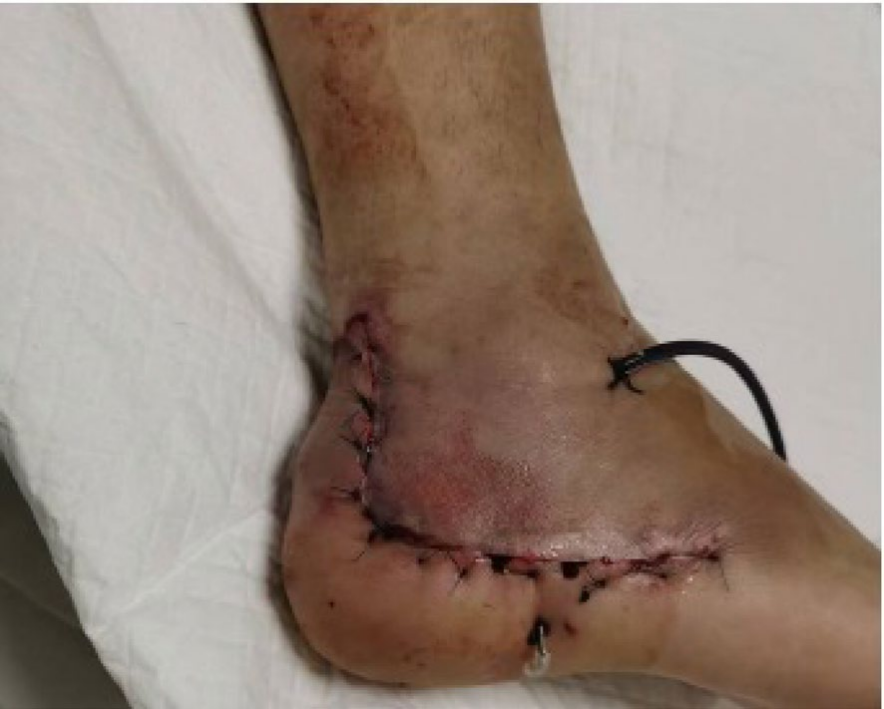
Allgöwer-Donati suture for incision closure

Examples of two types of closed wounds are shown in Figs. 6 and 7. After closing the wounds, two vials of tranexamic acid were diluted 1:1 with 10 mL of saline and injected into the closed wound through the drainage tube. The area was then clamped for 2 h to restore normal pressure. Sutures were removed 14 days after the operation.

### Indicators

#### Skin incision inflammatory reaction area

This study utilized a modified approach by Liu et al. [17] to quantitatively evaluate the area of inflammatory reaction zones surrounding the incision, which included erythema and edema with erosion or crusting. Standard electrocardiogram paper (1 mm² per grid) was placed over the inflammatory reaction area, and the paper was cut along the irregular edges to conform to the shape of the lesion. The total area was determined by counting the number of complete squares (mm^2^). In instances where multiple inflammatory reaction areas were present, measurements were taken individually and then summed to provide a total area [18]. Two senior orthopedic surgeons conducted all measurements independently, with each measurement being repeated three times to derive a mean value, thereby reducing potential human error.

#### Trend of skin temperature at the corner of L-shaped incisions

Skin temperature at the corners of L-shaped incisions was measured on the day before surgery and on postoperative days 1, 2, and 3, using an infrared thermometer. Measurements were consistently taken at 4 p.m., a time when fever peaks are typically observed, allowing for more pronounced temperature fluctuations and enhancing the detection of potential postoperative fever [19]. An experienced orthopaedic surgeon conducted the measurements at the same location to ensure consistency. Each measurement comprised three consecutive readings, and the final result was calculated as the average of these three values. This approach minimized random errors and enhanced the reliability of the outcomes.

#### Healing grade

Incision healing was evaluated based on established clinical criteria, which classified healing [20] into three grades. Grade A indicated excellent healing, characterized by an incision that exhibited no adverse reactions. Grade B represented suboptimal healing, where the incision remained non-purulent but displayed potential adverse reactions, such as erythema, edema, or exudate. Grade C indicated incision suppuration, necessitating either the separation of sutured incisions or incision drainage.

### Statistical analysis

The statistical analysis for this study utilized SPSS version 22.0. Continuous variables, such as age, wound closure time, inflammatory reaction area, and skin temperature at the incision corner at various time points, were reported as mean ± SD. Comparisons between groups were made using the independent samples t-test. Categorical variables, including gender, fracture classification, and incidence of incision complications, were expressed as counts (percentages), with intergroup comparisons conducted using the χ^2^ test or Fisher’s exact test. All hypothesis tests were two-tailed, with a significance threshold set at α = 0.05.

## Results

### Would closure time

All surgical procedures were completed successfully. To compare the wound closure times between the two groups, an independent samples t-test was conducted. The analysis revealed that the Stapler group had a significantly shorter closure time (10.89 ± 2.87 min) compared to the Allgöwer-Donati group (20.44 ± 2.01 min), with the difference being statistically significant (*P* < 0.001, Table 2).

**Table 2:** Wound closure time of the stapler group and the Allgower-Donati group $\left( {\overline{x} \pm s,\;{\mathrm{min}}} \right)$ (Comparison of wound closure time between groups)

Grouping	Number of cases	Suturing time	*P* value
The stapler group	28	10.89 ± 2.87	< 0.05
The Allgower-Donati group	32	20.44 ± 2.01	

### Inflammatory reaction area of the skin incision

The Stapler group showed mild local erythema around the incision site, along with sparse light brown scabbing. Similarly, the Allgöwer-Donati group exhibited mild erythema and light brown scabbing at the incision margins over a comparable area. Additionally, one patient in this group developed localized skin hyperpigmentation at the corner of the incision. Neither group showed signs of subcutaneous fat liquefaction or exposure of internal fixation. The statistical analysis revealed the inflammatory reaction area to be 35.34 ± 6.52 mm^2^ for the Stapler group and 36.93 ± 6.60 mm^2^ for the Allgöwer-Donati group, with no significant intergroup difference (*P* > 0.05, Table 3).

**Table 3:** Incision skin inflammatory reaction area following removal of sutures 2 weeks post-surgery of the stapler group and the Allgower-Donati group $\left( {\overline{x} \pm s,\;{\mathrm{mm}}^{{\mathrm{2}}} } \right)$ (Comparison of inflammatory reaction area between groups at 2 weeks post-surgery)

	Number of cases	Inflammatory reaction area	*P* value
The stapler group	28	35.34 ± 6.52	0.32
The Allgower-Donati group	32	36.93 ± 6.60	

### Skin temperature at the incision

On the day preceding surgery and on the first postoperative day, the skin temperature at the corners of the L-shaped incisions in both groups remained consistent (*P* > 0.05 for all comparisons, Table 4). Monitoring data from postoperative days 2 and 3 indicated that fluctuations in skin temperature were comparable between both groups (*P* > 0.05 for all comparisons, Table 4), with no significant differences observed in the microcirculation status of the incisions.

**Table 4:** Trend of skin temperature at the corner of L-shaped incisions of the stapler group and the Allgower-Donati group $\left( {\overline{x} \pm s,\;{^\circ }{\mathrm{C}}} \right)$ (Comparison of skin temperature changes at incision corners over time between groups)

	Number of cases	Before surgery	Day 1 after surgery	Day 2 after surgery	Day 3 after surgery
The stapler group	28	36.12 ± 0.43	36.63 ± 0.52	36.38 ± 0.47	36.08 ± 0.41
The Allgower-Donati group	32	36.11 ± 0.46	36.59 ± 0.49	36.35 ± 0.44	36.05 ± 0.39
t	–	0.35	0.45	0.75	0.53
P value	–	0.73	0.65	0.46	0.60

### Healing grade

All patients participated in a 6-month postoperative follow-up. The Stapler group achieved Grade A healing in 26 cases (92.9%) and Grade B healing in 2 cases (7.1%). One patient had minor bloody exudate postoperatively, which resolved with dressing changes. The Allgöwer-Donati group achieved Grade A healing in 27 cases (84.4%) and Grade B healing in 5 cases (15.6%). Two patients showed mild erythema and edema at the incision margins, but this did not affect healing. Fisher’s exact test showed no statistically significant difference in complication rates between the two groups (*P* = 0.428). Thus, there were no significant differences in healing grades or complication rates between the two suturing techniques.

## Discussion

The Allgöwer-Donati suturing technique effectively preserves blood supply to the incision margins, which helps to reduce the risk of postoperative complications and promotes better wound healing [18]. Previous research has shown that using the Allgöwer-Donati method for suturing surgical incisions in calcaneal fractures results in a high rate of favorable clinical outcomes, highlighting its clinical advantages [21, 22]. The success of this technique heavily depends on the surgeon’s skills. Key factors such as intraoperative suture tension control, the quality of tissue layer apposition, and the timing of drainage can significantly influence postoperative recovery. However, the Allgöwer-Donati method is relatively complex, necessitating longer suturing times and a high level of technical proficiency from the surgeon [22].

Skin closure using skin staplers has become widely accepted for closing incisions in various general surgical procedures [15, 23, 24]. The primary benefits of this method include its convenience and efficiency, which lead to significantly reduced wound closure times and shorter overall operation durations. This, in turn, minimizes the time patients spend under general anesthesia and lowers associated risks [25], while also decreasing the likelihood of incision infections. The use of skin staplers for incisions related to calcaneal fractures has been infrequently reported. However, their U-shaped design provides a larger tissue contact area, helping to distribute pressure evenly and reduce local peak pressure [26]. This mechanical distribution significantly lowers the risk of sutures cutting into delicate tissues during postoperative swelling, leading to less mechanical injury to the skin edges and a decreased rate of necrosis [27]. Furthermore, skin staplers minimize damage to vascular and neural structures at the incision margins [28]. Due to the limited vascular supply in the calcaneal region, this technique aids in preserving local microcirculation, which in turn promotes wound perfusion and healing. Furthermore, the use of skin staplers significantly minimizes reactions to suture knots [29], thus minimizing the risk of rejections associated with suture materials.

In comparative analyses, skin closure with skin staplers resulted in a similar inflammatory response area around the incision as observed in the Allgöwer-Donati group (Table 3). There was no statistically significant difference in the early postoperative inflammatory responses observed at the corners of L-shaped incisions, suggesting that both methods were equally effective in promoting local blood supply (Table 4). At the 6-month postoperative follow-up, the rates of Grade A healing and the incidence of complications were statistically comparable between the two groups. This similarity is likely due to the operational convenience and rapid closure provided by skin staplers, which effectively reduce tissue exposure time. Conversely, the Allgöwer-Donati suturing technique achieved favorable apposition and healing through its precise multi-layer suturing approach. A key advantage of using skin staplers is their high degree of procedural standardization, which reduces reliance on the operator’s skill, enhances suturing efficiency, improves healing quality, and demonstrates strong clinical applicability.

However, this study was conducted in an open design, which may introduce information bias. Additionally, the sample size was small and no prospective registration was performed. These limitations need to be further verified in subsequent studies. Advanced techniques such as intravascular fluorescence angiography and Doppler imaging can accurately measure microcirculatory blood flow during the healing process [30, 31], however, these methods were not utilized in this research. For the treatment of large L-shaped incisions in calcaneal fractures, options include the Allgower-Donati method, skin staplers, and the three-layer tight suture technique [18]. The latter technique, although effective, necessitates a relatively extended suturing period and was not utilized in this study. Another limitation of this study is that incision skin temperature was measured at a fixed time of day (4:00 p.m.) rather than at standardized intervals following surgery. Since the end times of surgeries varied, the postoperative measurement times were inconsistent among patients, potentially impacting the temperature readings. Future studies should measure temperature at fixed intervals after surgery to enhance the consistency of results.

Although skin staplers can expedite wound closure and help mitigate infection risk [32], they may also introduce significant artifacts in postoperative imaging, such as X-rays and CT scans, which could hinder the accurate interpretation of these images [33].

## Conclusions

Despite the limited sample size, this study found no evidence to suggest that the use of skin staplers for extended L-shaped incisions in calcaneal fractures compromises wound healing or increases long-term complication rates. This indicates that no significant differences in incision healing outcomes were observed between the two methods in this study. Skin staplers significantly reduced the operative time for wound closure (*P* < 0.05), enhancing suturing efficiency and potentially lowering the risk of infection. Given the methodological context of this unregistered trial, to further validate their clinical role, future studies with larger cohorts and prospective trial registration are warranted.

## Data Availability

The data and materials of this study will be stored at the Second Hospital of Shanxi Medical University and can be provided upon reasonable request. For specific access methods and procedures, please contact the corresponding author.

## References

[1] Razik A, Harris M, Trompeter A. Calcaneal fractures: Where are we now? Strateg Trauma Limb Reconstr. 2018;13(1):1–11. 10.1007/s11751-017-0297-3 PMC5862705 29052080

[2] Yu Q, Li Z, Li J, et al. Calcaneal fracture maps and their determinants. J Orthop Surg Res. 2022;17(1):39. 35062985 10.1186/s13018-022-02930-y PMC8780651

[3] Gougoulias N, McBride D, Maffulli N. Outcomes of management of displaced intra-articular calcaneal fractures. Surgeon. 2021;19(5):e222–9. 33262043 10.1016/j.surge.2020.10.003

[4] Gougoulias N, Khanna A, McBride DJ, et al. Management of calcaneal fractures: systematic review of randomized trials. Br Med Bull. 2009;92:153–67. 19734165 10.1093/bmb/ldp030

[5] Lu M, Cao S, Lu J, et al. Three dimensional analysis of factors affecting the prognosis of calcaneal fractures. J Orthop Surg Res. 2024;19(1):473. 39127669 10.1186/s13018-024-04975-7 PMC11316381

[6] Bibbo C, Ehrlich DA, Nguyen HML, et al. Low wound complication rates for the lateral extensile approach for calcaneal ORIF when the lateral calcaneal artery is patent. Foot Ankle Int. 2014;35(7):650–6. 24986898 10.1177/1071100714534654

[7] Choi WJ, Wang H, Wang RK. Optical coherence tomography microangiography for monitoring the response of vascular perfusion to external pressure on human skin tissue. J Biomed Opt. 2014;19(5):056003. 24810259 10.1117/1.JBO.19.5.056003 PMC4160975

[8] Niederstätter IM, Schiefer JL, Fuchs PC. Surgical strategies to promote cutaneous healing. Med Sci (Basel Switzerland). 2021;9(2):45. 10.3390/medsci9020045 PMC8293365 34208722

[9] Mutschler W. [Physiology and pathophysiology of wound healing of wound defects]. Der Unfallchirurg. 2012;115(9):767–73. 22935894 10.1007/s00113-012-2208-x

[10] Fadle AA, Khalifa AA, Shehata PM, et al. Extensible lateral approach versus sinus Tarsi approach for Sanders type II and III calcaneal fractures osteosynthesis: a randomized controlled trial of 186 fractures. J Orthop Surg Res. 2025;20(1):8. 39754179 10.1186/s13018-024-05345-z PMC11697837

[11] Li E, Zhang T, Ma Q, et al. Effect of modified Allgöwer–Donati suture technique on wound cosmetics in spinal surgery. Orthop Surg. 2022;14(4):678–85. 35179312 10.1111/os.13188 PMC9002073

[12] Shannon SF, Houdek MT, Wyles CC, et al. Allgöwer-Donati versus vertical mattress suture technique impact on perfusion in ankle fracture surgery: a randomized clinical trial using intraoperative angiography. J Orthop Trauma. 2017;31(2):97–102. 28129268 10.1097/BOT.0000000000000731

[13] Gafar M, M A E M, Mohammed AHM, Elseadawy AA et al. The effect of the primary wound closure technique in the surgical treatment of closed intra-articular calcaneal fractures. J Med Sci Res, 2024, 7(3).

[14] Magnani ND, Muresan XM, Belmonte G, et al. Skin damage mechanisms related to airborne particulate matter exposure. Toxicol Sci. 2016;149(1):227–36. 26507108 10.1093/toxsci/kfv230

[15] Lee J, Lee JE, Ryu JM, et al. The use of absorbable skin stapler in mastectomy does not increase the rate of surgical site infection. Annals Surg Treat Res. 2023;104(3):137–43. 36910562 10.4174/astr.2023.104.3.137 PMC9998960

[16] Al-Mubarak L, Al-Haddab M. Cutaneous wound closure materials: an overview and update. J Cutan Aesthetic Surg. 2013;6(4):178–88. 10.4103/0974-2077.123395 PMC3884880 24470712

[17] Liu DJ, Collaku A, Dosik JS. Skin irritation and sensitization potential of Fixed-Dose combination of diclofenac 1% and menthol 3% topical gel: results of two phase I patch studies. Volume 11. Drug Research, © Georg Thieme KG; 2017:119–26. 2. 10.1055/s-0042-118861 27887033

[18] Wu Y, Lv G, Sun H, Kaisar Y, Wang G. Effects of three-layer tight suturing and Allgöwer-Donati suturing on postoperative incision healing in calcaneal fractures. Chin J Orthop. 2019;39:579–84.

[19] Petretta R, McConkey M, Slobogean GP, et al. Incidence, risk factors, and diagnostic evaluation of postoperative fever in an orthopaedic trauma population. J Orthop Trauma. 2013;27(10):558–62. 23412504 10.1097/BOT.0b013e31828af4df

[20] Chhabra S, Chhabra N, Kaur A, et al. Wound healing concepts in clinical practice of OMFS. J Maxillofacial Oral Surg. 2017;16(4):403–23. 10.1007/s12663-016-0880-z PMC5628060 29038623

[21] Feng J, Hou J, Wang Y, et al. Application of Allgöwer-Donati suture in internal fixation of Schatzker type Ⅴand Ⅵ tibial plateau closed fractures. Zhongguo Xiu Fu Chong Jian Wai Ke Za Zhi = Zhongguo Xiufu Chongjian Waike Zazhi = Chinese. J Reparative Reconstr Surg. 2024;38(6):723–7. 10.7507/1002-1892.202401059 PMC11190689 38918194

[22] Shannon SF, Houdek MT, Wyles CC, et al. Allgöwer–Donati versus vertical mattress suture technique impact on perfusion in ankle fracture surgery: a randomized clinical trial using intraoperative angiography. J Orthop Trauma. 2017;31(2):97. 28129268 10.1097/BOT.0000000000000731

[23] Ranaboldo CJ, Rowe-Jones DC. Closure of laparotomy wounds: skin staples versus sutures. Br J Surg. 1992;79(11):1172–3. 1467895 10.1002/bjs.1800791122

[24] Grgić M, Ivkić M. Use of skin staplers in head and neck surgery: prospective clinical study. The J Otolaryngology. 2002;31(3):137–9. 10.2310/7070.2002.10785 12121014

[25] Stegmaier OC. Use of skin stapler in dermatologic surgery. J Am Acad Dermatol. 1982;6(3):305–9. 7068959 10.1016/s0190-9622(82)70020-9

[26] Chien WC, Tsai TF. Pressure and skin: a review of disease entities driven or influenced by mechanical pressure. Am J Clin Dermatol. 2024;25(2):261–80. 38159214 10.1007/s40257-023-00833-0

[27] Jia SS, Wang XC, Jiao Y, et al. [Research advances on skin wounds suturing techniques and their clinical application]. Zhonghua Shao Shang Za Zhi = Zhonghua Shaoshang Zazhi = Chin J Burns. 2021;37(11):1099–104. 10.3760/cma.j.cn501120-20200701-00334 PMC11917320 34794263

[28] Yang W, Peng Y, Li Y, et al. Arthroscopic repair of subscapularis tendon using a percutaneous continuous sewing machine–like suture technique through a single working portal. Arthrosc Tech. 2024;13(12):103151. 39780886 10.1016/j.eats.2024.103151 PMC11704904

[29] Byrne M, Aly A. The surgical suture. Aesthetic Surg J. 2019;39(Supplement2):S67–72. 10.1093/asj/sjz036 30869751

[30] Rother U, Lang W, Horch RE, et al. Pilot assessment of the angiosome concept by intra-operative fluorescence angiography after tibial bypass surgery. Eur J Vascular Endovascular Surgery: Official J Eur Soc Vascular Surg. 2018;55(2):215–21. 10.1016/j.ejvs.2017.11.024 29305093

[31] Mao Y, Mu J, Zhao J, et al. The comparative study of color doppler flow imaging, superb microvascular imaging, contrast-Enhanced ultrasound micro flow imaging in blood flow analysis of solid renal mass. Cancer Imaging: Official Publication Int Cancer Imaging Soc. 2022;22(1):21. 10.1186/s40644-022-00458-2 PMC9066849 35505388

[32] Korol E, Johnston K, Waser N, et al. A systematic review of risk factors associated with surgical site infections among surgical patients. PLoS ONE. 2013;8(12):e83743. 24367612 10.1371/journal.pone.0083743 PMC3867498

[33] Jungmann PM, Agten CA, Pfirrmann CW, et al. Advances in MRI around metal. J Magn Reson Imaging. 2017;46(4):972–91. 28342291 10.1002/jmri.25708

